# Laboratory tests of oviposition by the African malaria mosquito, *Anopheles gambiae*, on dark soil as influenced by presence or absence of vegetation

**DOI:** 10.1186/1475-2875-5-88

**Published:** 2006-10-12

**Authors:** Juan Huang, Edward D Walker, Philip E Otienoburu, Fred Amimo, John Vulule, James R Miller

**Affiliations:** 1 Department of Entomology, Michigan State University, East Lansing, MI 48823, USA; 2 Microbiology and Molecular Genetics, Michigan State University, East Lansing, MI 48824, USA; 3 Kenya Medical Research Institute, Kisumu, Kenya

## Abstract

**Background:**

Physical objects like vegetation can influence oviposition by mosquitoes on soil or water substrates. *Anopheles gambiae s. l*. is generally thought to utilize puddles over bare soil as its prime larval habitat and to avoid standing water populated with vegetation. In Kisian, Kenya near Kisumu, water often pools in grassy drainage areas both during and after periods of infrequent rains, when typical puddle habitats become scarce because of drying. This raised the question of whether *An. gambiae *has the behavioural flexibility to switch ovipositional sites when puddles over bare soil are unavailable.

**Methods:**

To test whether presence and height of grasses influenced oviposition, wild-caught gravid *An. gambiae s. l*. were offered paired choices between wet, bare soil and wet soil populated with mixed grasses or grasses of differing height. No-choice tests were also conducted by giving females either grassy soil or bare soil.

**Results:**

In choice tests, females laid four times more eggs on bare, wet soil than soil populated with grasses. However in no-choice tests, egg output was not significantly different whether grasses were present or not. Females laid significantly more eggs on soil populated with short grass than with medium, or tall grass.

**Conclusion:**

This work shows *An. gambiae s. l*. has the capacity to oviposit into grassy aquatic habitats when typical puddles over bare soil are unavailable. This knowledge will need to be considered in the design and implementation of programmes aimed at reducing malaria transmission by suppression of *An. gambiae s. l*. immatures.

## Background

Physical and chemical cues influence acceptance of ovipositional sites by mosquitoes [1-3]. Darkness and wetness are critical positive cues for *Anopheles gambiae *oviposition, while visual contrast strongly influences finding of prospective ovipositional sites [4,5]. Odours are not required for copious oviposition, however, they may increase egg output in some cases [6] and decrease it in others [7].

Physical objects like vegetation can influence oviposition on soil or water substrates [8-10]. Rice plants or glass rods inserted into seepage water reduced *Anopheles culicifacies *oviposition [8]. However, oviposition by *Anopheles hermsi *increased in accordance with the density of aquatic macrophytes (*Myriophylluym aquaticum*) up to 1,000 stems m^-2 ^[10]. In the field, *Anopheles funestus *breeds mainly in marshes and swamps that contain tall grasses and other plants [11]. *An. gambiae *is generally thought to utilize puddles over bare soil as its prime larval habitat [12-15] and to avoid standing water populated with vegetation. However, Muirhead-Thomson [16] reported that *An. gambiae *deposited eggs in rice fields at all stages of vegetative maturity. More recently, Fillinger *et al*. [17] have strongly challenged the idea that *An. gambiae *avoids habitats with emergent vegetation like grasses. They and Minakawa *et al*. [18] provide evidence that *An. gambiae *can be commonly found in association with grasses. However, it is unclear whether presence of larvae in grassy habitats results from hatch of eggs placed on and around wet grasses, or whether larvae were carried there by flowing water. The question addressed in the current laboratory study was whether *An. gambiae *has the behavioral flexibility to oviposit on and around wet grasses both when bare soil is and is not available.

## Materials and methods

### Mosquitoes and bioassay conditions

Experiments were performed in the Entomology laboratory of the Kenya Medical Research Institute (KEMRI) in Kisian, Kenya using feral females aspirated from houses near Kisian from 28 April to May 28, 2004, and from 15 April and 15 May 2005. These blood-fed females were kept for two days in cardboard cups (8 cm diam, 9 cm high) covered with wet cloth towels to provide moisture. Ten percent honey solution was also provided as an energy source. Gravid females were transferred into 60 × 60 × 60 cm white BugDorm-2 cages (MegaView Science Education Services Co., Taiwan) held on a lab bench near a bank of windows providing ambient nightlight. The response variable reported is total egg numbers. No detectable differences emerged in proportion of females retaining eggs. Although polymerase chain reaction was not performed on the wild-caught mosquitoes, previous samples from this area [4,14] contained > 80% *An. gambiae s. s*. Thus, results reported here represent *An. gambiae s. l*..

### Oviposition in response to wet soil populated with grass in 2004

#### Experiment 1: paired choice tests

Plugs of soil (8 cm height × 8 cm diam.) with associated grasses were collected outside the KEMRI compound near areas supporting *An. gambiae *larvae. These plugs were inserted into white cups of similar dimensions; they contained a mixture of Bermuda grass (*Cynodon dactylon*), sedge grass (*Scirpus steudneri*), and Rhode grass (*Chloris gayana*). Grass coverage of the soil ranged from 50 to 70%. Comparison cups were filled with soil taken from the same location but with no grass parts included. All the cups were saturated with tap water from a deep bore hole. A pair of cups with grass-covered vs. bare soil was offered to ca. 60 gravid females in a BugDorm-2 cage. Eleven replicates, each using a different batch of mosquitoes, were performed through time for choice tests.

#### Experiment 2: no-choice tests

The no-choice test was conducted (seven replicates) by providing 40 gravid females with a single ovipositional cup containing either grass-covered or corresponding bare soil. The following morning, grass stems were cut at their base and plants as well as soil were examined very thoroughly for the presence of eggs with the aid of a dissecting microscope.

### Oviposition in response to grasses of differing height in 2005

#### Experiment 3: paired choice tests

Soil plugs with grasses of differing height and corresponding bare soil were collected near the KEMRI compound. Over 90% of the soil was covered by grasses as compared to 50–70% in 2004. Grasses were divided into three height categories: short (1–2 cm), medium (13–16 cm), and tall (27–39 cm). It is acknowledged that species of grasses co-varied with the height categories such that effects cannot be attributed to height alone. Each grass category, paired with its control bare soil, was offered to 20 wild-caught gravid females.

#### Experiment 4: three-choice tests

Thirty gravid females were offered: short, medium, and tall grasses for oviposition. This test was replicated six times using different batches of females. Samples of the grasses used were dried and stored in layers of dry paper for subsequent species confirmation. Short grasses consisted of cape clover, *Trifolium burchellianum *(80%) and *Cynodon dactylon *(20%); medium grasses were *Cynodon dactylon *(50%) and *Scirpus steudneri *(50%); and tall grasses were *Chloris gayana*, the most dominant grass around Kisian.

### Statistical analysis

Differences in egg output were analysed by paired *t*-test in two-choice tests and no-choice tests. Differences in egg outputs in three-choice tests were calculated as a proportion of the total number of eggs laid in each cage and compared using 1-way analysis of variance (ANOVA) [19]. Means were separated by Tukey's significant difference (HSD).

## Results

### Oviposition in response to wet soil populated with grass in 2004

#### Experiment 1: paired choice tests

Wild-caught *An. gambiae *females deposited four times more eggs on bare wet soil than on soil covered by grasses (*t *= 4.0, *df *= 10, *P *= 0.002). Bare soil received 1,495 ± 224 eggs (mean ± SEM) while grass-covered soil received 353 ± 115 eggs.

#### Experiment 2: no-choice tests

**Figure 1:**
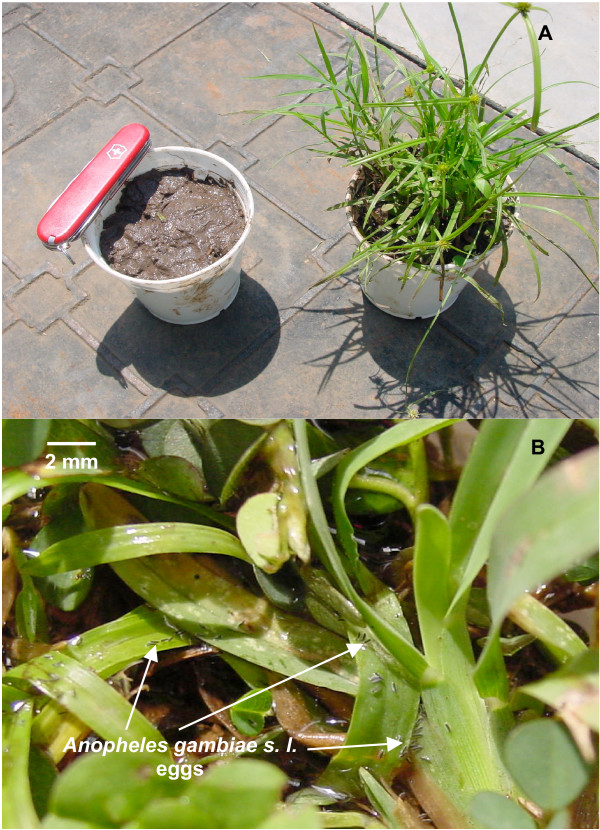
Examples of a paired choice test between wet bare soil and wet soil populated with grasses (A), and eggs laid by *Anopheles gambiae s. l*. on leaves of grass growing on wet soil (B).

Females laid 777 ± 73 eggs on bare soil compared to 573 ± 145 eggs on grass-covered soil (difference not significant: *t *= 1.0, *df *= 6, *P *= 0.35). Both in choice- and no-choice tests, some eggs were laid on the leaves or stems of grasses (Figure 1).

### Oviposition in response to grasses of differing height in 2005

#### Experiment 3: paired choice tests

**Figure 2:**
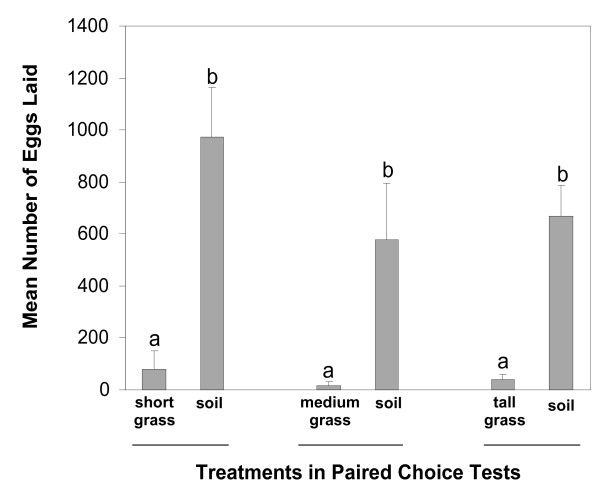
**Mean numbers of eggs by wild-caught *Anopheles gambiae s. l*. laid on grass-covered mud vs. its corresponding bare mud**. Within a choice test, bars topped with a common letter do not differ significantly at the 0.05 level (Tukey's HSD test). Error bars = S. E. M.

Females laid many more eggs on bare soil than the soil covered with grasses of differing heights (for short grass: *t *= 5.4, *df *= 5, *P *= 0.003; for medium grass: *t *= 2.6, *df *= 5, *P *= 0.05; for tall grass: *t *= 5.7, *df *= 5, *P *= 0.002) (Figure 2). However, 3 to 8% of the total eggs were laid on grass-covered soil when bare soil was nearby.

#### Experiment 4: three-choice tests

**Figure 3:**
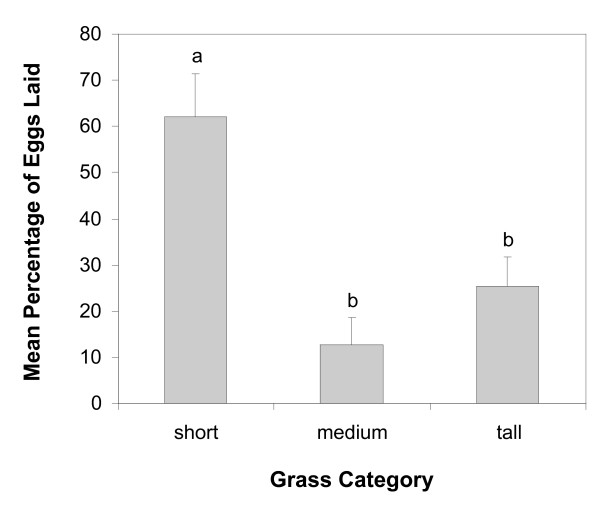
**Mean percentages of eggs laid by wild-caught *Anopheles gambiae s. l*. in a choice-test of mud covered by three different heights of grasses**. Bars topped with a common letter do not differ significantly at the 0.05 level (Tukey's HSD test). Error bars = S. E. M. The total number of eggs was 2393 for 180 female nights.

When no bare soil was present, more eggs were laid on the soil covered by short than by medium and tall grasses (*F *= 8.2; *df *= 2, 10; *P *= 0.008) (Figure 3).

## Discussion

When offered a choice, *An. gambiae s. l*. deposited many more eggs on bare, wet soil than grass-covered soil. Under laboratory conditions, *An. gambiae *usually touches the ovipositional substrate briefly before sitting on the mud to deposit eggs or hovers 5 to 10 cm above the puddle or mud while laying eggs (Huang et al. unpublished data). Short grasses were more acceptable than medium and tall grasses when no bare soil was present. Overall, egg abundance on grass-covered soil relative to bare soil decreased from 20 to less than 8% as the density of the grasses increased from 50–70% (in 2004) to 90% (in 2005). The density and height of grasses might act as a barrier precluding females from approaching sufficiently close to touch the wet substrate or to land. The speculation is offered that, if no bare soil is available, females might persist in exploring a wet grassy site and eventually alight and oviposit. In some cases, eggs appeared to have rained down upon grass leaves and axils (Figure 1). In the no-choice setting, females did not withhold eggs, but deposited copiously on whatever wet and dark resource was available. Provided they remain moist, eggs on grasses would develop [7] and, where there is a thin film of water, larvae would eclose and crawl [20,21] on the grasses, perhaps reaching water beneath. Alternatively, rain could wash eggs and larvae to pools [21].

This work confirms and extends previous claims that *An. gambiae *should be expected in rice fields [16] and other grassy habitats [17,18]. Females will oviposit on standing water, mud, moist sand [4], as well as mud with vegetation. This result further supports the view that *An. gambiae *is an ovipositional generalist [22] rather than a specialist. This malaria vector is flexible in ovipositional site selection, depending upon what ovipositional resources are available. Wet grassy zones could function as important secondary habitats when puddles over bare soil become scarce. This knowledge will need to be considered in the design and implementation of programmes aimed at reducing malaria transmission by suppression of *An. gambiae s. l*. immatures.

## Authors' contributions

JH designed and carried out all the experiments, analysed and interpreted data, as well as drafted and revised the manuscript. EDW was P. I. of the grant supporting this work; he participated in the conception of the study, data collection and interpretation, and revision of the manuscript. PEO helped collect grass samples and data. FA identified grass species. JV provided institutional support for this study. JRM participated in the study design, data collection and interpretation, as well as drafting and revising the manuscript, and submitting the manuscript. All authors read and approved the final manuscript.

## References

[B1] Takken W, Knols BGJ (1999). Odour-mediated behavior of Afrotropical malaria mosquitoes. Ann Rev Entomol.

[B2] Blackwell A, Johnson SN (2000). Electrophysiological investigation of larval water and potential oviposition chemo-attractants for *Anopheles gambiae s. s*. Ann Trop Med Parasit.

[B3] McCall P (2002). Chemoecology of oviposition in insects of medical and veterinary importance.

[B4] Huang J, Walker ED, Giroux PY, Vulule J, Miller JR (2005). Ovipositional site selection by *Anopheles gambiae*: influences of substrate moisture and texture. Med Vet Entomol.

[B5] Huang J, Walker ED, Vulule J, Miller JR (2006). The influence of darkness and visual contrast on oviposition by *Anopheles gambiae *in moist and dry substrates. Physiol Entomol.

[B6] Sumba LA, Guda TO, Deng AL, Hassanali A, Beier JC, Knols BGJ (2004). Mediation of oviposition site selection in the Africa malaria mosquito *Anopheles gambiae *(Diptera: Culicidae) by semiochemicals of microbial origin. Int J Trop Insect Sci.

[B7] Huang J, Miller JR, Chen S, Vulule J, Walker ED (2006). *Anopheles gambiae *(Diptera: Culicidae) oviposition in response to agarose media and cultured bacterial volatiles. J Med Entomol.

[B8] Russell PF, Rao TR (1942). On relation of mechanical obstruction and shade to oviposition of *Anopheles culicifacies*. J Exp Zool.

[B9] Chandler JA, Highton RB (1975). The succession of mosquito species (Diptera, Culicidae) in rice fields in the Kisumu area of Kenya, and their possible control. Bull Ent Res.

[B10] Orr BK, Resh VH (1992). Influence of *Myriophyllum aquaticum *cover on *Anopheles *mosquito abundance, oviposition, and larval microhabitat. Oecologia.

[B11] Clements AN (1999). The biology of mosquitoes.

[B12] Minakawa N, Mutero CM, Githure JI, Beier JC, Yan G (1999). Spatial distribution and habitat characterization of anopheline mosquito larvae in Western Kenya. Am J Trop Med Hyg.

[B13] Gimnig JE, Ombok M, Kamau L, Hawley WA (2001). Characteristics of larval anopheline (Diptera: Culicidae) habitats in Western Kenya. J Med Entomol.

[B14] Mutuku FMM, Alaii JA, Bayoh N, Gimnig JE, Vulule JM, Walker ED, Kabiru E, Hawley WA (2006). Distribution, description, and local knowledge of larval habitats of *Anohpeles gambiae s. l*. in a village in western Kenya. Am J Trop Med Hyg.

[B15] Mutuku FMM, Bayoh N, Gimnig JE, Vulule JM, Kamau L, Walker ED, Kabiru EW, Hawley WA (2006). Pupal habitat productivity of *Anopheles gambiae *complex mosquitoes in a rural village in Western Kenya. Amer J Trop Med Hyg.

[B16] Muirhead-Thomson RC (1945). Studies on the breeding places and control of *Anopheles gambiae *and *A. gambiae *var. *melas *in coastal districts of Sierra Leone. Bull Entomol Res.

[B17] Fillinger U, Sonye G, Killeen GF, Knols BGJ, Becker N (2004). The practical importance of permanent and semipermanent habitats for controlling aquatic stages of *Anopheles gambiae sensu lato *mosquitoes: operational observations from a rural town in western Kenya. Trop Med Int Health.

[B18] Minakawa N, Sonye G, Mogi M, Yan G (2004). Habitat characteristics of *Anopheles gambiae s. s*. larvae in Kenyan highland. Med Vet Entomol.

[B19] SAS Institute (1999). Guide for personal computers, Version 6.

[B20] Koenraadt CJM, Paaijmans KP, Githeko AK, Knols BGJ, Takken W (2003). Egg hatching, Larval movement and larval survival of the malaria vector *Anopheles gambiae *in desiccating environments. Malar J.

[B21] Miller JR, Huang J, Vulule J, Walker ED (2006). Life on the edge: African malaria mosquito larvae (*Anopheles gambiae*) are amphibious. Naturwissenschaften.

[B22] Holstein MH (1954). Biology of Anopheles gambiae.

